# Inflammatory Cytokine response in a cohort of patients carrying novel NLRP12 variants

**DOI:** 10.1186/1546-0096-13-S1-O23

**Published:** 2015-09-28

**Authors:** L Raganelli, G Prencipe, V Messia, I Caiello, FR Lepri, E Pisaneschi, F De Benedetti, A Insalaco

**Affiliations:** 1 Bambino Gesù Children Hospital, Pediatric Medicine-Rheumatology, Roma, Italy; 2 Bambino Gesù Children Hospital, Cytogenetics and Molecular Genetics, Rome, Italy

## Introduction

The NLRP12 related autoinflammatory disorder (NLRP12-RD) is a rare autosomal dominant disease, caused by mutations in the NLRP12 gene, a member of NLRs family involved in negative regulation of NF-kB pro-inflammatory pathways. At present, few cases have been reported, although novel NLRP12 variants have recently been described.

## Objective

To assess *ex vivo* the production of inflammatory cytokines, in patients carrying different NLRP12 variants not yet demonstrated as being associated to NLRP12-RD, in order to demonstrate the potential functional and pathogenic role of these variants.

## Materials and methods

30 children, carrying NLRP12 variants in heterozygosis, and 1 patient, carrying the F402L variant in homozygosis, were identified using Next Generation Sequencing [G39V (n=16), F402L (n=8), H304Y (n=3), T260M (n=2) and G448A (n=1)]; clinical information was collected for all patients. Patients with G39V were excluded from subsequent analysis, due to its high frequency both in our cohort (17.4%) as in the general population. Whole blood cultures from NLRP12 patients and Juvenile Idiopathic Arthritis (JIA) patients (n=10, pathological control) were stimulated *ex vivo* with TLRs ligands (LPS 1-10-100 ng/mL, Zymosan 10 μg/mL), with or without the further addition of ATP, for 5 or 22 hours. TNF-α, IL-1β and IL-6 were measured in supernatants by ELISA.

## Results

Among all stimuli used, we observed a clear inflammatory response in cells stimulated with 10mg/ml of Zymosan. Indeed, we found that the TNF-α release, after stimulation with Zymosan, was higherboth at 5 and 22 hours of incubationin all NLRP12 patients, although carrying different variants, compared to JIA patients. Moreover, we observed a trend for higher IL-6 levels released from a significant number of NLRP12 patients both at 5 and 22 hours of incubation. Interestingly, we did not find differences in the IL-1β levels measured in media from NLRP12 patients, compared with JIA patients. When we investigated parents, carrying the same NLRP12 variant, we did not find any differences in cytokines released in response to stimulation with TLRs ligands, compared with those obtained from age-matched healthy donors. Moreover, no association of a specific clinical phenotype with different NLRP12 variants, and a particularcytokine release signature was observed.

## Conclusion

We were not able to demonstrate a clear association of the investigated NLRP12 variants with the specific clinical phenotype or with the profile of pro-inflammatory cytokine released *ex vivo*. Other studies are needed before final conclusions on the pathogenic role of these NLRP12 variants can be drawn.

